# Increase water solubility of *Centellaasiatica* extract by indigenous bioenhancers could improve oral bioavailability and disposition kinetics of triterpenoid glycosides in beagle dogs

**DOI:** 10.1038/s41598-022-06967-1

**Published:** 2022-02-21

**Authors:** Tussapon Boonyarattanasoonthorn, Anusak Kijtawornrat, Phanit Songvut, Nitra Nuengchamnong, Visarut Buranasudja, Phisit Khemawoot

**Affiliations:** 1 Department of Physiology, Faculty of Veterinary Science, Chulalongkorn University, Bangkok, Thailand; 2 Laboratory of Pharmacology, Chulabhorn Research Institute, Bangkok, Thailand; 3 Science Laboratory Center, Faculty of Science, Naresuan University, Phitsanulok, Thailand; 4 Department of Pharmacology and Physiology, Faculty of Pharmaceutical Sciences, Chulalongkorn University, 254 Phayathai road, Pathumwan, Bangkok, 10330 Thailand; 5 Chakri Naruebodindra Medical Institute, Faculty of Medicine Ramathibodi Hospital, Mahidol University, Samutprakarn, 10540 Thailand

**Keywords:** Drug discovery, Medical research

## Abstract

A newly standardised extract of *Centella* *asiatica* (Centell-S) with better water solubility than the previous standardised extract of *C.* *asiatica* (ECa 233) was developed, and pharmacokinetic profiles of bioactive triterpenoids were investigated in beagle dogs. The test substances were administered via intravenous or oral administration with single and multiple doses for 7 days. The concentrations of major bioactive triterpenoids, including madecassoside, asiaticoside, madecassic acid, and asiatic acid, in biological samples were measured by liquid chromatography–tandem mass spectrometry. The dogs in this study showed good tolerability to all test substances, based on the physical appearance and blood chemistry 24 h after dosing. The major bioactive triterpenoids found in systemic blood circulation were madecassoside, asiaticoside, and asiatic acid; the concentration of these components ranged from 1 to 10,000 µg/L after intravenous administration of 1.0 mg/kg Centell-S. Oral administration of 10 and 20 mg/kg Centell-S generated approximately twofold higher plasma levels of both madecassoside and asiaticoside compared with equivalent doses of ECa 233. In addition, there was an accumulation of triterpenoid glycosides after multiple oral administrations of Centell-S for 7 days, while triterpenic acids showed little tendency for accumulation. Beagles had good tolerability to both standardised extracts of *C.* *asiatica*, and showed a similar pattern of bioactive triterpenoids to humans. Centell-S increased oral bioavailability of major triterpenoid glycosides and can be further developed into a phytopharmaceutical product.

## Introduction

*Centella* *asiatica* (Linn.) is commonly found in many areas around the world, especially in tropical areas of Asia, Africa, and the Americas^1–3^. This plant belongs to the Apiaceae family and has been developed into several phytopharmaceutical products. It has been used in alternative medicine to treat many diseases or lesions with good efficacy and safety^4–7^. ECa 233 is a commercial, standardised *C.* *asiatica* extract that has been developed by the Faculty of Pharmaceutical Sciences, Chulalongkorn University. This extract contains more than 80% w/w triterpenoid glycosides, with a constant ratio of madecassoside and asiaticoside (1.5 ± 0.5:1)^8,9^. ECa 233 has shown positive effects in a preclinical model of Alzheimer’s disease by reducing beta-amyloid^10^. Topical application of ECa 233 had a wound healing effect in a rat burn model^11^. In addition, an acute toxicological study in a mouse model showed normal vital signs after oral administration of up to 10 g/kg ECa 233. Moreover, in a subchronic toxicological study, mice given 10, 100, or 1000 mg/kg ECa 233 via oral administration for 90 days showed no significant differences in clinical signs compared with the control group^12^.

**Figure 1:**
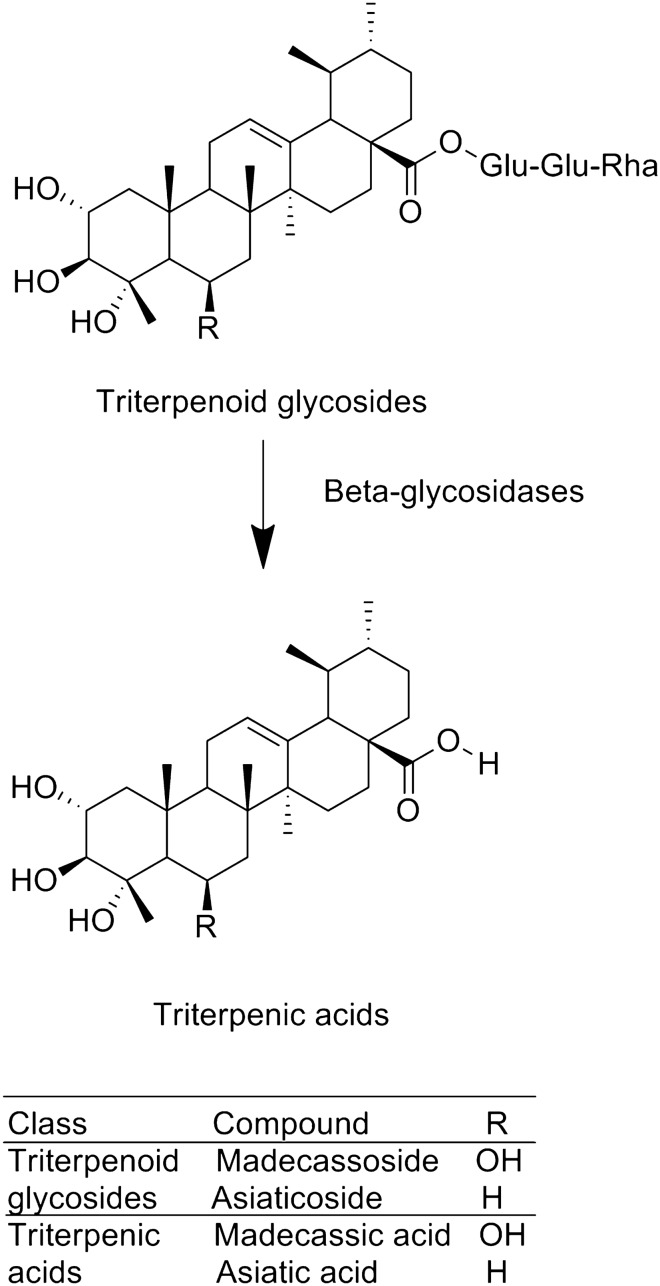
Chemical structure of bioactive triterpenoids in standardised extracts of *Centella* *asiatica*.

The triterpenoid glycosides of ECa 233 can be transformed to triterpenic acids, which are active metabolites, by anaerobic bacteria in the human digestive tract^13^ (Fig. 1). ECa 233 is sparingly soluble, and thus there is poor absorption after oral administration^14^. Recently, Siam Herbal Innovation Co., Ltd., developed a new standardised extract of *C.* *asiatica*, Centell-S, with similar components to ECa 233 but that is freely soluble in water. Centell-S may therefore provide greater oral bioavailability of triterpenoid glycosides compared with ECa 233, especially regarding absorption. For this reason, we investigated the pharmacokinetic profile of active triterpenoids in Centell-S and ECa 233, using beagle dogs as an animal model based on data from previous studies^14,15^. In our previous study, we examined the pharmacokinetics of ECa 233 and Centell-S, and demonstrated that rats showed considerable interspecies differences relative to humans. In rats, active metabolites were barely detected in plasma samples after the administration of ECa 233 and Centell-S. It is possible that rats have few microorganisms to transform parent triterpenoid glycosides to active triterpenic acids, while the human digestive tract has anaerobic bacteria that can transform the parent compounds into active metabolites^15^. This difference indicates that the results of pharmacokinetic studies from rats might have limited applications for human use.

Beagles represent a large animal species that is commonly used for pharmacokinetic studies in drug development, due to similarities in gastrointestinal anatomy and physiology compared to humans^16,17^. Moreover, the pharmacokinetics of both standardised extracts of *C.* *asiatica* (ECa 233 and Centell-S) have never been investigated in large animals. We therefore performed experiments in beagles to determine the pharmacokinetic profiles of major bioactive triterpenoids of *C.* *asiatica* in terms of absorption, biotransformation, and excretion. Dose selection was conducted based on safety and efficacy reports of standardised *C.* *asiatica* extract in rats. An appropriate dose selection in beagle dogs was calculated in accordance with the United States Food and Drug Administration (USFDA) guidance for industry in dose selection among mammals^18^, as well as a published paper from Hengjumrut et al*.*^19^. The results of this study may benefit the development of animal models in pharmacokinetic testing and the advancement of phytopharmaceutical products of *C.* *asiatica*.

## Materials and methods

### Chemicals

ECa 233 (lot number MRA 1118001, purity > 92.9%), Centell-S (lot number MA 0518001, purity > 86.7%), madecassoside (batch number HXJM-20190515, purity > 90.8%), and asiaticoside (batch number HXJM-20190515, purity > 91.1%) used for oral and intravenous administrations in this study were kindly provided by Siam Herbal Innovation Co., Ltd. The standardised extracts had 46% madecassoside and 41% asiaticoside as determined by liquid chromatography–tandem mass spectrometry (LC–MS/MS) analysis. All analytical standards were purchased from various sources: Chromadex Corp. for madecassoside (purity > 96.7%) and madecassic acid (purity > 97.5%); Sigma-Aldrich Inc. for asiaticoside (purity > 98.5%) and asiatic acid (purity > 97.0%); and Wako Pure Chemical Industries, Ltd., for internal standards, glycyrrhizin (purity > 90.0%), and glycyrrhetinic acid (purity > 98.0%).

### Animals

Eight healthy 8-month-old male beagles were obtained from the Chulalongkorn University Laboratory Animal Centre (CULAC), Chulalongkorn University. The dogs were randomly divided into two groups and housed in a controlled environment with temperature of 22 ± 2 °C, relative humidity 50% ± 20%, and a 12 h light/dark cycle. Food was provided twice a day, and water was available ad libitum. The dogs were fasted 12 h before the experiment, with free access to water. After the administration of the test substances, the dogs were placed in metabolic cages until 48 h post-administration. The animal experiments were approved by the Institutional Animal Care and Use Committee of the CULAC, Chulalongkorn University, Bangkok, Thailand (Protocol number 2073002, Approval date: February 21, 2020). All animal procedures were complied with the ethical principles and guidelines for the use of animals (National Research Council of Thailand, 2015). All animal experiments followed the ARRIVE reporting guidelines for documenting animal experiments.

### Pharmacokinetic study

For intravenous administration, there were four groups: vehicle, 0.46 mg/kg madecassoside, 0.41 mg/kg asiaticoside, and 1.00 mg/kg Centell-S. The test substance solutions were freshly prepared by using 20% v/v dimethyl sulfoxide/sterile water for injection and filtered through 0.22-µM polyvinylidene fluoride membrane (Merck Millipore, Ltd.) using a sterile technique, then a single dose of each test substances were injected via the cephalic or saphenous vein. For oral administration, there were four groups: 10 mg/kg ECa 233 (single dose), 20 mg/kg ECa 233 (repeated doses), 10 mg/kg Centell-S (single dose), and 20 mg/kg Centell-S (repeated doses). The test substance for oral administration was the powder of ECa233 and Centell-S which were provided by Siam Herbal Innovation Co., Ltd. The powder of both compounds were directly packed into the capsule without modification. One or two capsules containing the test substance were orally administered at 8:00 AM to the dogs following the experimental plan. Blood samples (2–3 mL) were collected from the cephalic or saphenous vein via a 22G intravenous catheter on day 1 and 7; at pre-dose; and at 0.08, 0.25, 0.5, 1, 2, 4, 8, and 24 h post-dose, and then put into pre-heparinised blood collection tubes. The blood samples were centrifuged at 5000*g* for 10 min at 4 °C to collect plasma and then stored at − 80 °C until analysis. Urine and faeces samples were collected in metabolic cages 0–24 and 24–48 h after the administration of test substances. The volume of urine and weight of faeces were recorded and all samples were stored at − 80 °C until analysis. All dogs were reused with a washed out period of two weeks, to allow complete excretion of bioactive triterpenoids. There were no residual bioactive triterpenoids detected by LCMS analysis. To determine the health status of the dogs, blood samples at 0 h pre-dose and 24 h post-dose were sent to the Small Animal Hospital, Faculty of Veterinary Science, Chulalongkorn University for blood chemistry tests.

### Sample preparation and method validation

All biological samples (plasma, urine, and faeces) were subjected to protein precipitation to extract the major triterpenoids from the samples. Two hundred microliters of methanol with two internal standards (glycyrrhizin and glycyrrhetinic acid) was added to 50 µL of the biological sample and mixed for 10 min. Then, the sample was centrifuged at 12,000*g* for 10 min at 4 °C, and 10 µL of supernatant was injected into the LC–MS/MS system. Faeces samples were homogenised by adding methanol, then the mixture was centrifuged at 1500*g* for 10 min at 4 °C to collect the supernatant. In the case of the concentration of the biological sample exceeded the linear calibration curve; blank matrices were used to dilute before protein precipitation. For method validation, all major components in the biological sample of dog were determined and validated according to the previous reports with little modification^15^. The method showed good specificity and linearity and no interference from other substances at the retention times of the targeted analytes and internal standards.

### Instrumentation

The LC–MS/MS system was set up according to a previous published method^15^. In brief, an LCMS-8060 coupled with a triple quadrupole mass spectrometer was controlled by LabSolution software version 5.86 (Shimadzu Corp.). A Synergi Fusion-RP C18 column (Phenomenex Inc.) maintained at 40 °C was used as the stationary phase. The analysis was run using absolute methanol and 0.2% formic acid in water as the mobile phase. The mobile phase used for gradient elution was 10% methanol at 0.0–0.5 min, increased to 90% methanol at 1.5 min and maintained until 3.0 min, then decreased to 10% methanol until 5.0 min at a flow rate of 0.5 mL/min. Multiple reaction monitoring with negative mode ionisation was performed to obtain the peak of madecassoside (973.40/503.30), asiaticoside (957.40/469.20), madecassic acid (503.25/437.15), asiatic acid (487.30/409.45), glycyrrhizin (821.25/350.90), and glycyrrhetinic acid (469.35/409.40). The calibration curves showed good linearity (R^2^ > 0.99), with a range from 1 to 10,000 µg/L for parent compounds (madecassoside and asiaticoside) and from 1 to 1000 µg/L for active metabolites (madecassic acid and asiatic acid). The lower limits of detection for both parent compounds and active metabolites were 1.00 µg/L.

For identification of minor compositions of Centell-S, an Agilent 6540 Q-TOF–MS spectrometer (Agilent Technologies Inc.) coupled with an Agilent 1260 Infinity Series High performance liquid chromatography system (Agilent Technologies Inc.). The separation was performed with a Luna C18 column, size 4.6 mm × 150 mm, 5 µm (Phenomenex Inc.) at a flow rate of 500 µL/min and the control temperature at 35 °C. The mobile phase A was water type I and B was acetonitrile. Both phases contained 0.1% (v/v) formic acid. The gradient elution mode started with 5% solvent B to 95% solvent B linear gradient within 30 min, and maintained at this ratio for 10 min after that post-run for 5 min. The injection volume was 10 µL. The operating parameters for MS detection were as follows: drying gas (N_2_) flow rate 10.0 L/min; temperature 350 °C; nebuliser pressure 30 psi; capillary 3500 V; skimmer 65 V; octupole RFV 750 V; and fragmentor voltage 250 V in negative mode and 100 V in positive mode. The mass range was set at *m/z* 100–1200 Da with a 250 ms/spectrum. The non-target MS/MS mode was set up at three collision energies of 10, 20, and 40 V. All data acquisition and analysis were controlled by MassHunter Data Acquisition Software version B.05.01 and MassHunter Qualitative Analysis Software B.06.00, respectively (Agilent Technologies Inc.). Analysis of each sample was performed both in positive and negative ionisation modes including non-targeted MS/MS mode to provide abundant information for structural identification.

### Data analysis

To evaluate significant differences between the pre- and post-dose of the test substances to the health status of dogs, the biochemical data were compared by using Student’s *t*-test. PK solution software version 2.0 (Summit Research Services) was used to analyse pharmacokinetic parameters by non-compartmental analysis. C_max_ and T_max_ were directly determined from plasma concentration–time curves. AUC_0-24_ was calculated using the trapezoidal rule and extrapolated to time infinity by the equation AUC_0-inf_ = AUC_0–24_ + (C_last_/k_el_), where C_last_ is the last observed plasma concentration after dosing and k_el_ is the elimination rate constant. MRT was calculated as AUMC_0-inf_/AUC_0-inf_, where AUMC_0-inf_ is the area under the first moment concentration–time curve. Vd was equal to dose/C_0_, where C_0_ is the plasma concentration at time zero and CL was calculated as dose/AUC_0-inf_. The terminal elimination half-life was determined by dividing 0.693 by k_el_. Absolute bioavailability was calculated as (AUC_p.o._/DOSE_p.o._)/(AUC_i.v._/DOSE_i.v._) × 100. Relative bioavailability was calculated from (AUC_CTS_/DOSE_CTS_)/(AUC_ECa_/DOSE_ECa_) × 100. Before performing the statistical analysis in SPSS version 22.0 (IBM Corp.), the Shapiro–Wilk test was used to determine whether the data were normally distributed. A paired Student’s *t* test or Wilcoxon signed-rank test was used to determine significant differences of pharmacokinetic parameters between the groups. The percent recovery of triterpenoid glycosides and triterpenic acids were calculated using the total amount of the test substance found in urine or faeces divided by the given dose. All data are expressed as the mean ± standard deviation except for Tmax and half-life, which were expressed as median (IQR). Differences were considered significant at *p* < 0.05.

## Results

### Plasma concentration–time profiles

All of the dogs used in the study tolerated the test substances. Blood samples were collected from each dog pre- and post-administration of madecassoside, asiaticoside, ECa 233, and Centell-S. Then, the plasma was sent for clinical laboratory testing to determine haematology, kidney, and liver functions. There were no significant differences in the measured biochemical parameters between pre-dose and post-dose in any group (Supplementary Table S1).

**Figure 2:**
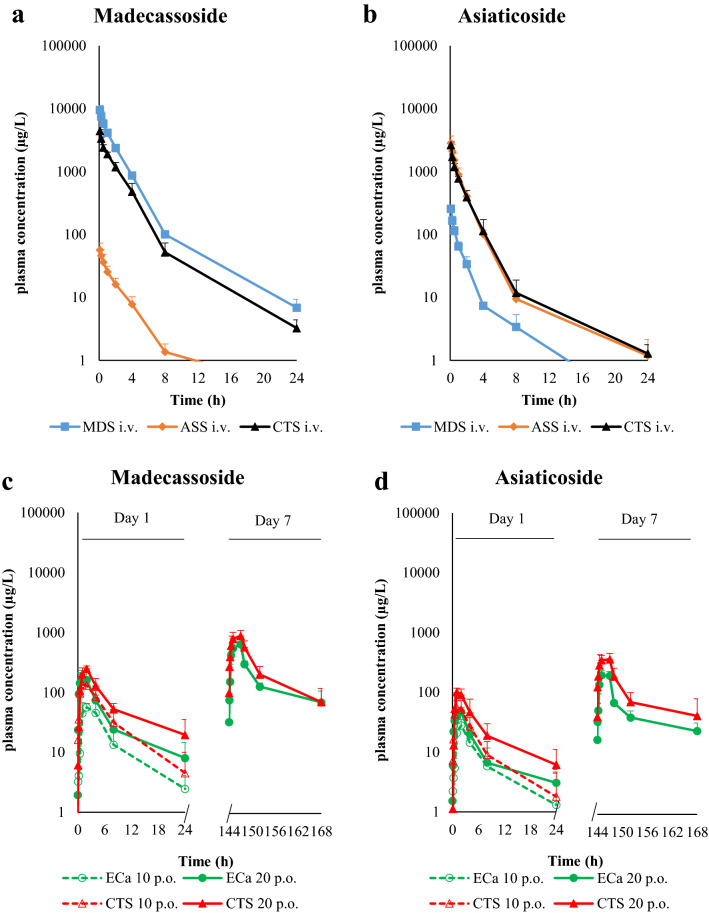
Plasma concentration–time profiles of madecassoside and its conversion from asiaticoside (**a**) and asiaticoside and its conversion from madecassoside (**b**) after intravenous administration of pure compounds and Centell-S, and madecassoside (**c**) and asiaticoside (**d**) after oral administration of ECa 233 and Centell-S. Data are presented as the mean ± standard deviation (n = 4).

**Figure 3:**
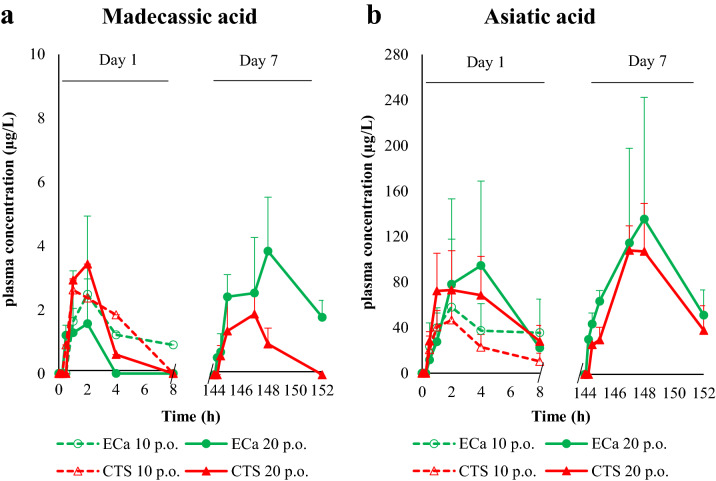
Plasma concentration–time profiles of madecassic acid (**a**) and asiatic acid (**b**) after oral administration of ECa 233 and Centell-S at 10 and 20 mg/kg. Data are presented as the mean ± standard deviation (n = 4).

The plasma concentration–time profile of triterpenoid glycosides after single and multiple administrations of intravenous and oral doses of madecassoside, asiaticoside, ECa 233, and Centell-S are shown in Fig. 2. For intravenous administration of 0.46 mg/kg madecassoside, the plasma concentration of this substance reached a maximum of approximately 10,000 µg/L, then declined to 10 µg/L 24 h after dosing (Fig. 2a). For intravenous administration of 0.41 mg/kg asiaticoside, the plasma concentration reached a maximum of approximately 3000 µg/L, then declined to 10 µg/L 8 h after dosing (Fig. 2b). For intravenous administration of 1 mg/kg Centell-S, the plasma concentration of madecassoside and asiaticoside showed similar pharmacokinetic patterns to the pure substances. Remarkably, intravenous administration of pure madecassoside generated a significant amount of asiaticoside and vice versa; this finding may indicate bidirectional in vivo interconversion between the two triterpenoid glycosides. A single oral administration of 20 mg/kg ECa 233 showed maximal madecassoside and asiaticoside plasma concentrations that were approximately twofold higher than oral administration of 10 mg/kg ECa 233 (Fig. 2c,d). After 7 days of multiple oral administration of 20 mg/kg ECa 233, the maximum plasma concentration of madecassoside was approximately 1.5 fold greater than the first day of administration. The plasma concentrations of madecassoside and asiaticoside showed similar pharmacokinetic patterns between oral administration of ECa 233 and Centell-S. However, oral administration of Centell-S was associated with maximal plasma concentrations that were approximately twofold higher compared with equivalent doses of ECa 233. The plasma concentration–time profile of the active metabolites, madecassic acid and asiatic acid, after single and multiple oral administration of ECa 233 and Centell-S are shown in Fig. 3. These active metabolites were detected in the plasma from 30 min to 8 h after administration. The asiatic acid concentration was approximately tenfold higher than madecassic acid. There was no statistically significant difference in the triterpenic acid level between oral administration of ECa 233 and Centell-S.

### Pharmacokinetic parameters

The main pharmacokinetic parameters of the parent compounds and active metabolites after administration of all test substances are listed in Tables 1, 2 and 3. After intravenous injection of the pure compound, the maximum plasma concentration of madecassoside was 10,023 ± 2021 µg/L. After intravenous Centell-S injection, there was a significantly lower maximum plasma concentration of madecassoside (4546 ± 122 µg/L). In addition, the volume of distribution (Vd) and clearance (CL) of madecassoside in the Centell-S group showed a significantly higher value than pure madecassoside. The half-life of both triterpenoid glycosides was approximately 4–5 h after intravenous administration of all test compounds (Table 1). For oral administration of Centell-S, the time to reach the maximum plasma concentration (T_max_) of madecassoside and asiaticoside was observed 0.5–2 h after oral administration. The absolute bioavailability of triterpenoid glycosides after single oral dosing was approximately 0.6–1.2% for Centell-S and 0.3–0.6% for ECa 233. Noticeably, the maximum plasma concentration (C_max_) of madecassoside, asiaticoside, and asiatic acid after oral administration of 20 mg/kg Centell-S was approximately twofold higher than the group that received oral administration of 10 mg/kg Centell-S. Furthermore, the accumulation of asiaticoside, as indicated by the C_max_, showed a statistically significant difference (*p* < 0.05) between single and multiple doses of Centell-S (Table 2). Oral administration of ECa 233 generated a lower C_max_ and a delayed T_max_ for both triterpenoid glycosides compared with Centell-S. The relative oral bioavailability of both triterpenoid glycosides in Centell-S group was approximately 200% of ECa 233 group. The Vd and CL of madecassoside and asiaticoside after single and multiple oral dosing showed higher values than intravenous administration of Centell-S. Repeated oral administration of ECa 233 and Centell-S prolonged the mean residence time (MRT) of both madecassoside and asiaticoside. The major active metabolite found in beagles was asiatic acid, and repeated oral administration increased the level of asiatic acid. After intravenous administration of madecassoside, asiaticoside, and Centell-S, most of the parent triterpenoid glycosides were excreted via urine within 24 h after dosing. Madecassoside and asiaticoside from oral ECa 233 and Centell-S were mainly excreted via faeces. Negligible amounts of the active metabolites, madecassic and asiatic acids, were found in the excreta of beagles after intravenous or oral dosing of all test compounds (Supplementary Table S2). The volume and weight of excreta which was used to calculate percent recovery of bioactive triterpenoids after receiving the test compounds were shown in Supplementary Table S3.

**Table 1:** Pharmacokinetic parameters of madecassoside and asiaticoside after intravenous administration of 0.46 mg/kg madecassoside, 0.41 mg/kg asiaticoside, or 1.00 mg/kg Centell-S.

Pharmacokinetic parameters	Madecassoside		Asiaticoside	
	MDS 0.46 mg/kg i.v	CTS 1.00 mg/kg i.v	ASS 0.41 mg/kg i.v	CTS 1.00 mg/kg i.v
C_max_^a^ (µg/L)	10,023 ± 2021	4546 ± 122*	3630 ± 1322	3351 ± 1439
AUC_0-24_^a^ (µg.h/L)	15,662 ± 1927	7518 ± 1026*	3207 ± 829	2912 ± 318
AUC_0-inf_^a^ (µg.h/L)	15,705 ± 1938	7538 ± 1021*	3218 ± 835	2922 ± 316
MRT^a^ (h)	8.05 ± 0.99	8.23 ± 0.84	8.83 ± 3.31	9.53 ± 1.59
Vd^a^ (L/kg)	0.18 ± 0.05	0.38 ± 0.10*	0.88 ± 0.30	1.08 ± 0.38
CL^a^ (L/h/kg)	0.03 ± 0.01	0.06 ± 0.01*	0.14 ± 0.04	0.14 ± 0.01
Half-life^b^ (h)	3.88 (0.40)	3.88 (0.57)	5.04 (2.32)	4.95 (1.12)

*C*_*max*_ maximum plasma concentration, *AUC*_*0-24*_ area under the plasma concentration–time curve from time 0–24 h, *AUC*_*0-inf*_ area under the plasma concentration–time curve from time 0-infinity, *MRT* mean residence time, *Vd* volume of distribution, *CL* clearance, *MDS* madecassoside, *ASS* asiaticoside, *CTS* Centell-S.

^a^Data are expressed as mean ± SD; ^b^Data are expressed as median (IQR); *p < 0.05 for significant differences.

**Table 2:** Pharmacokinetic parameters of madecassoside and asiaticoside (a) and madecassic acid and asiatic acid (b) after oral administration of 10 and 20 mg/kg Centell-S as a single dose or repeated doses.

Pharmacokinetic parameters	Madecassoside			Asiaticoside		
	CTS 10 mg/kg p.o	CTS 20 mg/kg p.o. Day 1	CTS 20 mg/kg p.o. Day 7	CTS 10 mg/kg p.o	CTS 20 mg/kg p.o. Day 1	CTS 20 mg/kg p.o. Day 7
**(a)** **Parent** **compounds** **(triterpenoid** **glycosides)**						
C_max_^a^ (µg/L)	144.23 ± 38.17	250.83 ± 32.27*	368.45 ± 70.76	60.50 ± 16.78	107.05 ± 20.04*	137.25 ± 12.00*
T_max_^b^ (h)	1.00 (0.38)	2.00 (0.25)	1.50 (1.13)	0.75 (0.75)	1.00 (0.25)	1.25 (1.50)
AUC_0-24_^a^ (µg.h/L)	949 ± 340	1637 ± 354*	2201 ± 666	324 ± 143	610 ± 282	704 ± 261
AUC_0-inf_^a^ (µg.h/L)	994 ± 381	2168 ± 1149	2494 ± 899	354 ± 182	708 ± 357	1356 ± 1383
Relative bioavailability^c^ (%)	214	188	N/A	172	225	N/A
MRT^a^ (h)	9.23 ± 10.54	21.68 ± 15.22	18.55 ± 7.14	11.88 ± 7.37	16.23 ± 4.91	41.08 ± 40.34
Vd^a^ (L/kg)	0.26 ± 0.15	0.49 ± 0.17	N/A	1.17 ± 0.64	1.94 ± 0.77	N/A
CL^a^ (L/h/kg)	0.03 ± 0.01	0.03 ± 0.02	N/A	0.16 ± 0.08	0.16 ± 0.10	N/A
Half-life^b^ (h)	5.25 (3.55)	10.14 (11.23)	9.79 (7.69)	4.44 (4.52)	8.68 (5.00)	17.44 (26.85)
**(b)** **Active** **metabolites** **(triterpenic** **acids)**						
C_max_^a^ (µg/L)	2.65 ± 0.35	3.40 ± 0.55	1.88 ± 0.88	52.93 ± 18.86	102.63 ± 20.65*	128.30 ± 15.15
T_max_^b^ (h)	1.00 (0.00)	2.00 (0.00)	1.50 (1.13)	1.50 (1.00)	3.00 (2.25)	3.00 (2.00)
AUC_0-24_^a^ (µg.h/L)	9.50 ± 0.99	10.60 ± 1.53	6.58 ± 3.61	226 ± 127	586 ± 290	1000 ± 358
AUC_0-inf_^a^ (µg.h/L)	9.55 ± 0.92	10.70 ± 1.69	6.63 ± 3.66	326 ± 33	699 ± 221	1725 ± 1206

*C*_*max*_ maximum plasma concentration, *T*_*max*_ time to reach C_max_, *AUC*_*0-24*_ area under the plasma concentration–time curve from time 0–24 h, *AUC*_*0-inf*_ area under the plasma concentration–time curve from time 0-infinity, *MRT* mean residence time, *Vd* volume of distribution, *CL* clearance, *CTS* Centell-S, *N/A* not available.

^a^Data are expressed as mean ± SD; ^b^Data are expressed as median (IQR); ^c^Relative bioavailability was calculated as (AUC_CTS_/DOSE_CTS_)/(AUC_ECa_./DOSE_ECa_) × 100; *p < 0.05 for significant differences.

**Table 3:** Pharmacokinetic parameters of madecassoside and asiaticoside (a) and madecassic acid and asiatic acid (b) after oral administration of 10 and 20 mg/kg ECa 233 as a single dose or repeated doses.

Pharmacokinetic parameters	Madecassoside			Asiaticoside		
	ECa 10 mg/kg p.o	ECa 20 mg/kg p.o. Day 1	ECa 20 mg/kg p.o. Day 7	ECa 10 mg/kg p.o	ECa 20 mg/kg p.o. Day 1	ECa 20 mg/kg p.o. Day 7
**(a)** **Parent** **compounds** **(triterpenoid** **glycosides)**						
C_max_^a^ (µg/L)	64.58 ± 18.85	176.48 ± 64.19	237.70 ± 40.28	30.15 ± 6.69	49.25 ± 12.23	62.70 ± 9.67
T_max_^b^ (h)	2.00 (0.75)	2.00 (0.25)	2.00 (0.25)	1.50 (1.00)	2.00 (0.25)	1.50 (1.00)
AUC_0-24_^a^ (µg.h/L)	413 ± 211	958 ± 329	1314 ± 253	172 ± 81	269 ± 36	308 ± 39
AUC_0-inf_^a^ (µg.h/L)	465 ± 195	1153 ± 450	1857 ± 792	206 ± 73	314 ± 58	519 ± 310
Absolute bioavailability^c^ (%)	0.29	0.37	N/A	0.64	0.48	N/A
MRT^a^ (h)	20.05 ± 12.21	19.68 ± 14.41	27.38 ± 15.70	23.35 ± 16.99	19.27 ± 8.85	39.43 ± 34.80
Vd^a^ (L/kg)	0.55 ± 0.40	0.47 ± 0.28	N/A	3.78 ± 1.23	2.05 ± 0.69	N/A
CL^a^ (L/h/kg)	0.03 ± 0.02	0.03 ± 0.02	N/A	0.14 ± 0.05	0.17 ± 0.03	N/A
Half-life^b^ (h)	9.36 (6.90)	8.68 (8.81)	17.06 (12.07)	14.59 (10.96)	11.61 (4.69)	15.59 (15.60)
**(b)** **Active** **metabolites** **(triterpenic** **acids)**						
C_max_^a^ (µg/L)	2.50 ± 2.43	2.00 ± 0.60	3.63 ± 1.29	70.93 ± 51.30	97.83 ± 71.13	166.70 ± 96.69
T_max_^b^ (h)	2.00 (0.25)	1.00 (0.50)	2.50 (3.00)	1.50 (1.63)	4.00 (0.50)	3.00 (2.25)
AUC_0-24_^a^ (µg.h/L)	9.23 ± 9.66	5.07 ± 2.89	35.30 ± 10.71	504 ± 459	662 ± 363	1172 ± 652
AUC_0-inf_^a^ (µg.h/L)	9.25 ± 9.68	5.10 ± 2.94	35.30 ± 10.71	632 ± 466	679 ± 378	1335 ± 951

*C*_*max*_ maximum plasma concentration, *T*_*max*_ time to reach C_max_, *AUC*_*0-24*_ area under the plasma concentration–time curve from time 0–24 h, *AUC*_*0-inf*_ area under the plasma concentration–time curve from time 0-infinity, *MRT* mean residence time, *Vd* volume of distribution, *CL* clearance, *ECa* ECa 233, *N/A* not available.

^a^Data are expressed as mean ± SD; ^b^Data are expressed as median (IQR); ^c^Absolute bioavailability was calculated as (AUC_p.o._/DOSE_p.o._)/(AUC_i.v._/DOSE_i.v._) × 100; * *p* < 0.05 for significant differences.

### LC ESI-QTOF-MS/MS analysis

**Figure 4:**
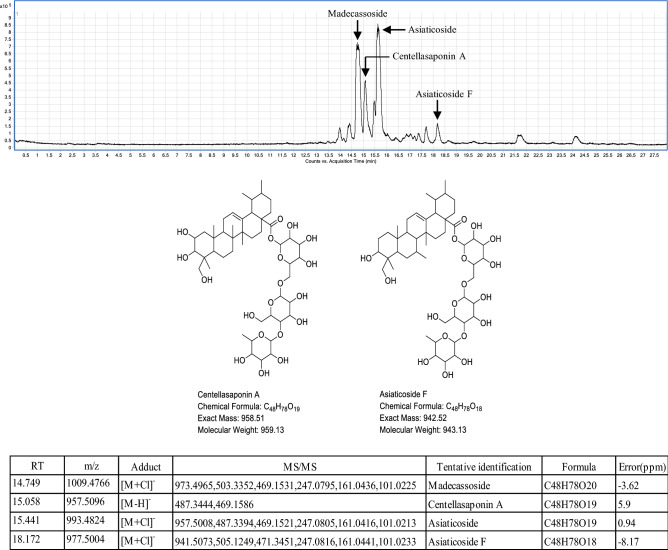
Total ion chromatogram of Centell-S at concentration 1 mg/mL operated in negative mode (top), the proposed structure of compounds found in Centell-S (middle), and MS/MS data of their tentative identification (bottom).

Regarding the composition of Centell-S assessed by LC ESI-QTOF-MS/MS analysis, two major and two minor compositions were found (Fig. 4). The first major peak was detected at m/z 1009.4766 [M+Cl]^−^ with a retention time of 14.749 min and was deduced to be a chloride adduct of madecassoside. The second major peak was identified at m/z 993.4824 [M+Cl]^−^ with a retention time of 15.441 min and was deduced to be a chloride adduct of asiaticoside. The standards of madecassoside and asiaticoside were used to confirm the major peaks that showed the identical molecular weight and retention time. The first minor peak was detected at a retention time of 15.058 min with a molecular weight 957.5096 g/mol which was proposed as a hydrogen abduct [M−H]^−^ of centellasaponin A (exact molecular weight: 958.51 g/mol). The second minor peak was identified at a retention time of 18.172 min with a molecular weight 977.5004 g/mol which was considered as a chloride adduct [M+Cl]^−^ of asiaticoside F (exact molecular weight: 942.52 g/mol). However, the confirmation of these two minor peaks remained undetermined because of the absence of commercial standards.

QTOF-MS/MS was used to confirm the identity of the saponin compound in Centell-S. The pseudo-molecular ion can be seen in chloride adduct form in negative mode and ammonium adduct in positive mode. Madecassoside and asiaticoside were identified and confirmed the mass fragmentation pattern and retention time with authentic compounds. Centellasaponin A and asiaticoside had the same molecular weight and almost the same MS/MS fragmentation pattern. When considering Xlog P, centellasaponin A (XlogP = 0.59) was more hydrophilic than asiaticoside (XlogP = 0.73), so the retention time of centellasaponin A was ahead of asiaticoside. The next compound with m/z 977.5004 [M+Cl]^−^ had 16 Da less than centellasaponin A and was proposed as asiaticoside F.

## Discussion

Drug absorption can be altered by many factors, the most common of which is water solubility. A test compound with limited water solubility usually has low absorption, resulting in low oral bioavailability^20^. In general, triterpenoid glycosides, especially madecassoside and asiaticoside, have large molecular sizes and contain a sugar moiety that reduces absorption through the intestinal membrane^13^. Moreover, efflux transporters, for example, P-glycoprotein could efflux the absorbed madecassoside return to the gastrointestinal tract^21^. The standardised extract of ECa 233 has low oral bioavailability due to its limited water solubility^14,15^. To improve the solubility of ECa 233, Centell-S was developed. This test compound is freely soluble in water and, similar to ECa 233, its major components are madecassoside and asiaticoside. The absolute oral bioavailability of both triterpenoid glycosides was determined, and Centell-S had higher value by approximately twofold. Two minor components, centellasaponin A and asiaticoside F were detected in Centell-S that might act as bioenhancers. Previously, our group has reported the minor components of ECa 233, and found that one centellasaponin in ECa 233 could act as a bioenhancer^22^. The improved bioavailability of Centell-S might be due to the improvement in the water solubility of Centell-S.

In this study, we aimed to determine the pharmacokinetic profile of bioactive triterpenoids in Centell-S compared with ECa 233. All experimental animals showed good tolerability to the test substances after oral and intravenous administration. The results implied that the test substances, especially Centell-S, are safe in animals, a finding that is in line with our previous reports in standardised extract of *C.* *asiatica*^14,15,19,23^. After intravenous administration of Centell-S, the plasma concentrations of madecassoside and asiaticoside were slightly lower than after pure compound administration. The potential mechanism could involve a minor component found in Centell-S that might act as a bioenhancer and increase the tissue distribution of triterpenoid glycosides. This was correlated with a study by Yurdakok-Dikmen et al. reporting that *C.* *asiatica* contains some bioenhancers that can facilitate tissue distribution^24^. Therefore, a higher Vd could be observed for madecassoside and asiaticoside of Centell-S. Improved tissue distribution could result in a lower plasma level of the triterpenoid glycosides, since the blood volume in the vascular compartment is accounts for only 5–10% of the total body volume. Moreover, asiaticoside showed a higher Vd than madecassoside, which also implies that asiaticoside may distribute to tissues better than madecassoside. This phenomenon relates to the partition coefficient of asiaticoside (XlogP = 0.1), which is higher than that of madecassoside (XlogP = − 1.2). Therefore, asiaticoside has greater lipophilic properties than madecassoside prefers residing in tissue compartments. This result is well correlated with a study on ECa 233 in rats, which showed the distribution of asiaticoside to many tissues, including the lungs, heart, brain, skin, and liver, after intravenous administration of ECa 233^14^. We accidentally detected a small peak of asiaticoside generated by intravenous administration of pure madecassoside or vice versa in experimented animals. In addition, there was no peak of asiaticoside found in the fresh preparation and blank plasma of madecassoside group or vice versa. This result was consistent with our report from 2018, which was a comparative pharmacokinetic study between the pure compound and the standardised extract of *C.* *asiatica* in rats by Hengjumrut et al*.*^19^. These results suggested that bidirectional interconversion between madecassoside and asiaticoside could be detected in both rats and dogs. This bidirectional interconversion could prolong systemic exposure of the two glycosides and might increase pharmacodynamic activities of these triterpenoid glycosides in vivo. To add hydroxyl group into asiaticoside structure, it might be needed some oxidative enzymes, e.g., CYP isoforms. In contrary, conversion of madecassoside to asiaticoside might require reduction processes in order to remove hydroxyl group from madecassoside structure. Further study to determine responsible enzymes and appropriate conditions of this bidirectional interconversion is required.

Increasing the Centell-S or ECa 233 oral dose from 10 to 20 mg/kg in beagles increased the C_max_ and AUC of madecassoside and asiaticoside by approximately twofold. After oral administration of Centell-S or ECa 233, the parent triterpenoid glycosides were metabolised to triterpenic acids by anaerobic bacteria in the intestine, a phenomenon that has been reported in many studies^13,15,25^. Asiatic acid might be converted from asiaticoside by hydrolytic degradation of the sugar moiety^26^. In this study, asiatic acid was the main active metabolite detected in the plasma of dogs from 0.5 to 8.0 h after oral administration of Centell-S or ECa 233. This finding correlates well with results from humans, namely that asiatic acid is a major active metabolite in plasma after oral administration of 250–500 mg capsules of ECa 233^15^. The similar pharmacokinetic profiles of bioactive triterpenoids in humans and dogs might imply that these species have a similar gut microbiota, which can biotransform triterpenoid glycosides into triterpenic acids^27,28^. This result was different from the pharmacokinetic study of ECa 233 in rats, which did not detect any active metabolites in the plasma and internal organs of rats^14^. After increasing the concentration of a single oral dose of Centell-S from 10 to 20 mg/kg, the C_max_ of asiatic acid increased approximately twofold. Furthermore, 7 days of repeated Centell-S administration significantly changed the C_max_ of asiatic acid compared with the first day. Asiatic acid seems to be the major active metabolite in both ECa 233 and Centell-S in dogs, and many studies have determined that asiatic acid has therapeutic effects for the treatment of various diseases^29–31^. In our study, madecassoside and asiaticoside were mainly excreted via the urine after intravenous administration of all test substances. However, after oral administration of ECa 233 and Centell-S, the main route for madecassoside and asiaticoside excretion was via the faeces. A certain amount of asiatic acid was found in the faeces within 24 h after oral administration of Centell-S and ECa 233, suggesting that the gut microbiota had the ability to hydrolyse the sugar moiety of the parent triterpenoid glycosides of *C.* *asiatica* to active triterpenic acid. This result agrees with a study by Songvut et al. who reported that active triterpenic acids are eliminated via the faecal route^32^.

## Conclusion

Beagles had good tolerability to the standardised extract of *C.* *asiatica*, and showed similar pharmacokinetic profiles of bioactive triterpenoids to humans. Improved water solubility of Centell-S led to higher oral bioavailability of madecassoside and asiaticoside than ECa 233 in beagle dogs. The pharmacokinetic results could benefit future phytopharmaceutical product development from *C.* *asiatica*.

## Supplementary Information

Supplementary Information.

## Abbreviations

ALT: Alanine aminotransferase
ASA: Asiatic acid
ASS: Asiaticoside
AST: Aspartate aminotransferase
AUC: Area under the curve
CL: Total clearance
C_max_: Maximum plasma concentration
Centell-S: Standardised extract of *Centella* *asiatica* with improved water solubility
ECa 233: Standardised extract of *Centella* *asiatica* with poor water solubility
MDA: Madecassic acid
MDS: Madecassoside
MRT: Mean residence time
T_max_: Time to reach maximum plasma concentration
Vd: Volume of distribution
