# Supplementary material for: Increase water solubility of Centellaasiatica extract by indigenous bioenhancers could improve oral bioavailability and disposition kinetics of triterpenoid glycosides in beagle dogs
Source: Sci Rep. 2022 Feb 21;12:2909. doi: 10.1038/s41598-022-06967-1 (PMC8861063; doi:10.1038/s41598-022-06967-1)
Supplement: Supplementary file 1 — Supplementary Information. [file 41598_2022_6967_MOESM1_ESM.pdf]

Supplementary data

Table S1 Physical and biochemical profiles of experimental beagles.

Biochemical parameters		Experimental groups									
		Control	MDS 0.46 mg/kg i.v.	ASS 0.41 mg/kg i.v.	CTS 1.00 mg/kg i.v.	ECa 10 mg/kg p.o.	ECa 20 mg/kg p.o. Day 1	ECa 20 mg/kg p.o. Day 7	CTS 10 mg/kg p.o.	CTS 20 mg/kg p.o. Day 1	CTS 20 mg/kg p.o. Day 7
Physical appearance	Pretreatment	Normal	Normal	Normal	Normal	Normal	Normal	Normal	Normal	Normal	Normal
	Post-treatment	Normal	Normal	Normal	Normal	Normal	Normal	Normal	Normal	Normal	Normal
Body weight	Pretreatment	11.67 ± 1.21	12.25 ± 1.31	11.60 ± 1.52	11.48 ± 1.27	11.27 ± 1.43	11.52 ± 1.75	11.37 ± 1.72	10.76 ± 1.35	11.60 ± 1.43	11.11 ± 1.38
	Post-treatment	11.83 ± 1.11	12.36 ± 1.25	11.87 ± 1.49	11.62 ± 1.33	11.39 ± 1.34	11.71 ± 1.66	11.52 ± 1.76	10.87 ± 1.29	11.82 ± 1.35	11.23 ± 1.29
White blood cell (x 10^3/µL)	Pretreatment	14.24 ± 2.29	14.87 ± 3.17	12.34 ± 2.17	15.10 ± 1.17	10.86 ± 2.99	11.45 ± 2.33	11.80 ± 3.81	11.33 ± 3.56	10.56 ± 2.88	11.11 ± 5.32
	Post-treatment	12.90 ± 4.93	11.29 ± 2.64	10.17 ± 3.01	12.73 ± 1.56	10.43 ± 3.31	11.05 ± 1.92	11.06 ± 3.02	10.79 ± 2.83	10.43 ± 3.25	11.65 ± 4.60
Red blood cell (x 10^6/µL)	Pretreatment	6.18 ± 0.45	6.11 ± 0.40	6.05 ± 0.57	5.74 ± 0.65	6.27 ± 0.56	6.20 ± 0.41	6.05 ± 0.23	6.48 ± 0.46	6.25 ± 0.58	6.27 ± 0.33
	Post-treatment	5.46 ± 0.90	5.39 ± 0.86	5.23 ± 0.88	5.82 ± 0.61	5.43 ± 0.24	5.94 ± 0.53	5.68 ± 0.39	6.11 ± 0.54	6.20 ± 0.52	5.96 ± 0.53
Hemoglobin (g/dL)	Pretreatment	14.0 ± 0.8	13.4 ± 0.7	13.6 ± 0.9	13.1 ± 1.0	13.9 ± 0.7	14.5 ± 1.1	13.9 ± 0.4	13.9 ± 0.6	13.9 ± 1.1	13.9 ± 0.5
	Post-treatment	12.4 ± 1.8	12.2 ± 1.6	12.1 ± 1.7	13.0 ± 1.1	12.3 ± 0.5	13.3 ± 1.4	13.2 ± 0.5	13.0 ± 0.9	13.2 ± 0.9	13.0 ± 0.9
Platelet (x 10^3/µL)	Pretreatment	320 ± 52	353 ± 65	328 ± 49	326 ± 70	281 ± 84	265 ± 59	266 ± 62	305 ± 77	279 ± 48	275 ± 62
	Post-treatment	215 ± 131	236 ±110	233 ± 109	333 ± 66	237 ± 104	258 ± 52	244 ± 56	243 ± 100	272 ± 65	257 ± 61
Blood urea nitrogen (mg/dL)	Pretreatment	11.8 ± 3.6	15.5 ± 2.0	11.8 ± 2.0	12.2 ± 2.8	8.7 ± 1.5	11.9 ± 1.8	10.3 ± 1.6	8.9 ± 1.4	10.2 ± 1.9	8.7 ± 1.3
	Post-treatment	15.6 ± 2.7	13.6 ± 1.5	11.4 ± 2.1	11.3 ± 2.8	7.8 ± 1.9	11.2 ± 1.4	11.8 ± 1.3	11.0 ± 1.0	9.6 ± 1.6	9.6 ± 2.0
Creatinine (mg/dL)	Pretreatment	0.6 ± 0.1	0.6 ± 0.1	0.6 ± 0.1	0.5 ± 0.1	0.5 ± 0.0	0.5 ± 0.1	0.6 ± 0.1	0.5 ± 0.1	0.5 ± 0.1	0.5 ± 0.1
	Post-treatment	0.5 ± 0.1	0.6 ± 0.1	0.5 ± 0.1	0.6 ± 0.1	0.5 ± 0.1	0.5 ± 0.1	0.5 ± 0.1	0.5 ± 0.1	0.5 ± 0.1	0.5 ± 0.1
AST (U/L)	Pretreatment	32 ± 0	33 ± 6	25 ± 4	28 ± 1	28 ± 3	25 ± 5	28 ± 2	23 ± 2	24 ± 3	27 ± 3
	Post-treatment	23 ± 3	27 ± 6	30 ± 10	24 ± 3	25 ± 3	24 ± 4	29 ± 0	23 ± 2	22 ± 4	27 ± 1
ALT (U/L)	Pretreatment	57 ± 18	54 ± 11	55 ± 5	46 ± 5	43 ± 8	42 ± 9	43 ± 6	39 ± 6	48 ± 9	44 ± 6
	Post-treatment	50 ± 13	51 ± 7	53 ± 4	44 ± 4	42 ± 9	45 ± 9	43 ± 8	40 ± 6	48 ± 7	43 ± 5

AST: aspartate transaminase; ALT: alanine transaminase; MDS: madecassoside; ASS: asiaticoside; CTS: Centell-S; ECa: ECa 233. Data are expressed as mean ± SD, (n = 4); * $p < 0.05$ for significant differences. Decimal numbers were reported according to laboratory standard of Small Animal Hospital, Faculty of Veterinary Science, Chulalongkorn University.

Table S2 Percentage recovery of madecassoside, asiaticoside, madecassic acid, and asiatic acid in excreta of all experimental groups.

Percent recovery	Experimental groups															
	MDS 0.46 mg/kg i.v.		ASS 0.41 mg/kg i.v.		CTS 1 mg/kg i.v.		ECa 10 mg/kg p.o.		ECa 20 mg/kg p.o. Day 1	ECa 20 mg/kg p.o. Day 7	ECa 20 mg/kg p.o. Day 7	CTS 10 mg/kg p.o.		CTS 20 mg/kg p.o. Day 1	CTS 20 mg/kg p.o. Day 7	CTS 20 mg/kg p.o. Day 7
	0-24 h	24-48 h	0-24 h	24-48 h	0-24 h	24-48 h	0-24 h	24-48 h	0-24 h	0-24 h	24-48 h	0-24 h	24-48 h	0-24 h	0-24 h	24-48 h
Urine																
Madecassoside	90.79 ± 13.99	1.16 ± 0.29	4.55 ± 3.07	< 1.00	34.94 ± 3.98	1.01 ± 1.45	5.78 ± 1.42	1.46 ± 0.23	12.52 ± 2.89	16.10 ± 6.03	0.84 ± 0.26	6.32 ± 1.62	0.58 ± 0.18	16.84 ± 3.42	11.06 ± 3.86	1.78 ± 0.89
Asiaticoside	< 1.00	< 1.00	30.27 ± 14.87	< 1.00	7.90 ± 1.24	< 1.00	0.81 ± 0.23	< 1.00	1.46 ± 0.36	3.33 ± 1.40	< 1.00	0.95 ± 0.25	< 1.00	2.61 ± 0.51	2.06 ± 0.79	< 1.00
Feces																
Madecassoside	4.59 ± 3.60	2.69 ± 0.89	8.34 ± 1.09	1.98 ± 1.55	18.55 ± 4.29	3.25 ± 4.15	45.83 ± 47.63	< 1.00	40.28 ± 11.79	41.91 ± 10.22	26.70 ± 10.84	43.45 ± 13.15	< 1.00	43.39 ± 27.98	69.28 ± 10.09	18.06 ± 12.58
Asiaticoside	< 1.00	< 1.00	24.07 ± 7.71	6.76 ± 5.95	24.79 ± 7.66	4.29 ± 5.39	21.15 ± 21.77	< 1.00	16.59 ± 7.77	14.83 ± 5.23	10.69 ± 5.91	16.91 ± 9.32	< 1.00	19.63 ± 6.93	24.77 ± 13.19	10.27 ± 7.98
Madecassic acid	< 1.00	< 1.00	< 1.00	< 1.00	< 1.00	< 1.00	< 1.00	< 1.00	< 1.00	< 1.00	< 1.00	< 1.00	< 1.00	< 1.00	0.52 ± 0.33	< 1.00
Asiatic acid	< 1.00	< 1.00	< 1.00	< 1.00	< 1.00	< 1.00	1.07 ± 1.11	< 1.00	6.42 ± 17.42	8.98 ± 8.27	< 1.00	0.90 ± 0.20	< 1.00	< 1.00	7.36 ± 9.30	< 1.00

MDS: madecassoside; ASS: asiaticoside; CTS: Centell-S; ECa: ECa 233. Data are expressed as mean ± SD, (n = 4).

Table S3 Real volume and weight of excreta in all experimental groups.

Real volume/weight	Experimental groups																	
	Vehicle		MDS 0.46 mg/kg i.v.		ASS 0.41 mg/kg i.v.		CTS 1 mg/kg i.v.		ECa 10 mg/kg p.o.		ECa 20 mg/kg p.o. Day 1	ECa 20 mg/kg p.o. Day 7	ECa 20 mg/kg p.o. Day 7	CTS 10 mg/kg p.o.		CTS 20 mg/kg p.o. Day 1	CTS 20 mg/kg p.o. Day 7	CTS 20 mg/kg p.o. Day 7
	0-24 h	24-48 h	0-24 h	24-48 h	0-24 h	24-48 h	0-24 h	24-48 h	0-24 h	24-48 h	0-24 h	0-24 h	24-48 h	0-24 h	24-48 h	0-24 h	0-24 h	24-48 h
Urine (mL)	85 ± 18	91 ± 12	121 ± 42	98 ± 56	88 ± 52	75 ± 19	92 ± 20	91 ± 15	108 ± 31	95 ± 39	82 ± 9	82 ± 40	149 ± 27	95 ± 20	94 ± 19	136 ± 56	123 ± 25	129 ± 55
Feces (g)	99 ± 28	105 ± 19	75 ± 48	105 ± 17	119 ± 56	123 ± 34	106 ± 53	108 ± 52	48 ± 24	66 ± 41	196 ± 87	119 ± 20	129 ± 54	93 ± 11	83 ± 28	84 ± 53	144 ± 32	113 ± 35

MDS: madecassoside; ASS: asiaticoside; CTS: Centell-S; ECa: ECa 233. Data are expressed as mean ± SD, (n = 4).
